# The ties to unbind: age-related differences in feature (un)binding in working memory for emotional faces

**DOI:** 10.3389/fpsyg.2014.00253

**Published:** 2014-04-21

**Authors:** Didem Pehlivanoglu, Shivangi Jain, Robert Ariel, Paul Verhaeghen

**Affiliations:** School of Psychology, Georgia Institute of TechnologyAtlanta, GA, USA

**Keywords:** hyper-binding, memory binding, working memory, aging, pupillary response, emotion

## Abstract

In the present study, we investigated age-related differences in the processing of emotional stimuli. Specifically, we were interested in whether older adults would show deficits in unbinding emotional expression (i.e., either no emotion, happiness, anger, or disgust) from bound stimuli (i.e., photographs of faces expressing these emotions), as a hyper-binding account of age-related differences in working memory would predict. Younger and older adults completed different *N*-Back tasks (side-by-side 0-Back, 1-Back, 2-Back) under three conditions: match/mismatch judgments based on either the identity of the face (identity condition), the face’s emotional expression (expression condition), or both identity and expression of the face (both condition). The two age groups performed more slowly and with lower accuracy in the expression condition than in the both condition, indicating the presence of an unbinding process. This unbinding effect was more pronounced in older adults than in younger adults, but only in the 2-Back task. Thus, older adults seemed to have a specific deficit in unbinding in working memory. Additionally, no age-related differences were found in accuracy in the 0-Back task, but such differences emerged in the 1-Back task, and were further magnified in the 2-Back task, indicating independent age-related differences in attention/STM and working memory. Pupil dilation data confirmed that the attention/STM version of the task (1-Back) is more effortful for older adults than younger adults.

## INTRODUCTION

Working memory is the workspace of the mind, where passive storage and active manipulation/transformation of information engage in dynamic interplay (Baddeley and Hitch, 1974; Miyake and Shah, 1999; Kane et al., 2001). An effective working memory system is crucial for high-level performance in a multitude of cognitive tasks, perhaps because an effective working memory system depends on the efficient implementation of a host of basic cognitive control operations that are likely involved in most, if not all aspects of the cognitive system. For instance, significant relations have been demonstrated between fluid intelligence and working memory capacity, and between spatial and language abilities and working memory (e.g., Kyllonen, 1996; Engle et al., 1999; Conway et al., 2002; Kemper et al., 2003).

Given the centrality of the working memory construct, it is not surprising that there is a growing literature on the effects of aging on its capacity and dynamics. The brunt of the research shows that working memory capacity declines with advancing adult age. Small age differences are already found in the performance of short-term memory (STM) tasks that do not require much cognitive control or attentional resources, such as digit span tasks, but age-related deficits in working memory, as measured by tasks such as reading span, listening span, or operation span, are demonstrably larger. In a meta-analysis compiling a total of 123 studies from 104 papers, Bopp and Verhaeghen (2007) found that older adults’ capacity in simple STM span tasks was 92% that of the capacity of younger adults; their capacity on true working memory tasks, however, was only 74% that of younger adults. This decline in working memory capacity has consequences for complex aspects of cognition: on average, working memory capacity explains 52% of the age-related variance in episodic memory performance, 63% of the age-related variance in spatial abilities, and 72% of the age-related variance in reasoning abilities; STM, in contrast, explains 9% or less of the age-related variance (Verhaeghen, 2014).

One particular determinant of working memory capacity that has consistently been found to be age-sensitive is the availability of information that has left focus of attention and needs to be retrieved after a bout of intermittent processing. Our lab and others have studied this topic using variations on the *N*-Back task – the task we will also use here (e.g., Oberauer, 2002; Verhaeghen and Basak, 2005; Chen and Li, 2007; Vaughan et al., 2008; Verhaeghen, 2012; Zhang et al., 2012). In an *N*-Back task, subjects indicate whether the stimulus currently on the screen matches the stimulus presented *N* positions back. The typical finding in our experiments (but see Kirchner, 1958; Dobbs and Rule, 1989) is that older and younger adults are equally (and highly) accurate in the 1-Back version of the task, but that age differences in accuracy consistently emerge when *N* > 1. We would argue that 1-Back tasks primarily reflect the workings of attention, with a possible STM component as well (i.e., subjects have to retain information passively over a very brief duration – 2 s in our experiment). A 2-Back version adds a true working memory component to the task, in the form of intervening items that need to be processed. The finding then echoes other reports (e.g., Bopp and Verhaeghen, 2007) that older adults show small or not deficits in STM tasks, but substantial deficits in working memory tasks. Additionally, in the present study, we will include a 0-Back condition where two stimuli are shown side-by-side, to test and/or control for perceptual deficiencies in older adults.

The finding of an age-related deficit in the working memory component, but not the attentional/STM component of working memory leads to an obvious question: can this result be reduced to a deeper deficit in a known process, or is this aspect of working memory – retrieval of items stored outside immediate attention – a cognitive primitive that is especially age-sensitive? This is the research question we will address in the present paper. To foreshadow: the cognitive primitive we will investigate is association, more precisely, the binding of features into objects and the concomitant unbinding of objects into features; the objects we will use, for reasons explained below, are faces displaying emotional expressions (presenting emotion and identity into a compound percept). Additionally, we will use a physiological measure (pupil dilation) to independently verify the commitment of attentional resources throughout the task. As a side effect, the use of emotional stimuli will allow us to investigate the claims of an age-related positivity effect in a domain where such effects have rarely been investigated, namely that of working memory.

One popular hypothesis to explain age-related differences in working memory functioning is the hypothesis of an age-related associative deficit. The claim is that older adults fail to efficiently and/or effectively link individual features of a learning episode together; one consequence is a particular difficulty in forming or retrieving the associations or bindings between the features of objects, compared to maintaining the single features in memory (Chalfonte and Johnson, 1996; Naveh-Benjamin et al., 2004; Cowan et al., 2006). The associative-binding theory has fared extremely well in the domain of episodic memory (e.g., Old and Naveh-Benjamin, 2008), but evidence from the domain of working memory have been decidedly mixed.

In support of the associative-deficit hypothesis, Cowan et al. (2006), in a delayed match-to-sample task, did indeed find evidence that older adults were especially poor at detecting binding changes. Importantly, this deficit was not due to age-related differences in attentional resources per se, because the decline was evident even when the set size of the array was reduced to be well below capacity limits. Other researchers, in contrast, have failed to discover evidence for an associative deficit in working memory, concluding instead that aging is governed by a rather general deficit in working memory functioning. For instance, in three change-detection experiments, Brockmole et al. (2008) found overall lower working memory performance in older adults, but no specific age-related deficit emerged in a feature-both condition. Importantly, in this study, older adults were more affected by increases in set size than younger adults were, suggesting that the age-related decline in working memory performance is tied to a fundamental change in capacity rather than to associative deficits. With a very different task – a repetition-detection paradigm – Bopp and Verhaeghen (2009) found, in line with the Brockmole et al. (2008) findings, that the necessity to bind content to context decreased performance in both age groups to the same degree.

In the present paper, we approach the binding problem from a different angle, inspired by a recent study that found evidence – in episodic memory – of the existence of the third possible empirical outcome, namely that older adults are better at associative binding than younger adults. That is, Campbell et al. (2010) found that when to-be-remembered stimuli (in this case: line drawings) were paired with stimuli explicitly labeled as irrelevant (in this case: words) older participants showed better memory for the drawing–word conjunction than younger adults did. The authors labeled this phenomenon hyper-binding, and see it as predicated on deficient inhibitory functioning. Under this hypothesis, older adults are unable to inhibit the irrelevant information, which therefore gets encoded (and maintained) along with the relevant information. The net result is a functional shrinkage in the capacity of working memory.

So far, age-related hyper-binding has not been demonstrated in working memory. Clearly, the three papers cited above report either no evidence for a binding deficit in old age, or the opposite of hyper-binding, that is, a binding deficit. It is also clear, however, that the standard set-up of experiments into the associative deficit hypothesis might not be easily conducive to elicit the phenomenon of age-related hyper-binding. One potential issue is that in classical association experiments (which often use artificial compound stimuli composed of random combinations of abstract features, e.g., Wheeler and Treisman, 2002) feature binding typically comes at a cost: remembering the compound object is more difficult than remembering the individual features. Working memory experiments reporting age-related binding deficits as evidenced by age by condition interactions could then simply be instantiations of a complexity or difficulty effect: age differences are “naturally” larger in tasks yielding lower performance, that is, in this case, the both condition (e.g., Cerella, 1985).

In the present study, we reversed this situation by using naturally occurring and ecologically valid compound objects that, by all accounts, are easier to remember than at least some of their constituent features. More specifically, we capitalized on an interesting finding in the face recognition literature, namely that when participants are confronted with a face showing a natural emotion, recognition of facial identity (i.e., who is this person?) typically occurs faster and with higher accuracy than recognition of the emotional expression (e.g., Ganel and Goshen-Gottstein, 2004; Martens et al., 2010). At the same time, matching on both identity and emotional expression typically occurs no faster or more accurately than matching on identity alone (e.g., Soto and Wasserman, 2011). These findings suggest that identity of faces is processed easily, maybe even automatically, whereas extraction of emotional expression is a much slower, potentially effortful process. (Note that there is some controversy over whether these two types of judgments – identity or emotional expression – are independent; Bruce and Young, 1986; or interrelated; e.g., Ganel and Goshen-Gottstein, 2004. This question, however, is irrelevant to our current study, which concerns only the relative efficiency of these processes.) This particular quirk of the cognitive system makes emotional face recognition particularly well suited to investigate the hyper-binding hypothesis. If older adults indeed encode more of the stimulus than strictly needed for the task, the age-related deficit in performance would be larger in conditions in which only the emotional expression (a single feature) needs to be remembered compared to memory for the whole stimulus (i.e., identity bound with emotional expression), because older adults would be less inclined or less capable to extract and remember only the relevant feature. (Note that the age-related deficit for memory for facial identity (another single feature) should likely match that for memory of the whole stimulus, given that identity extraction typically comes at no cost.)

One added advantage of these stimuli lies in their emotional nature: it has often been claimed that the valence of emotions inherent in experimental stimuli moderates age-related differences in cognition. Our study allows us to investigate some of these claims explicitly, in a domain where these have been rarely researched. For instance, socioemotional selectivity theory (Carstensen et al., 1999) states that a shrinking time perspective leads older adults to focus on positive information rather than negative information – the so-called positivity bias. There are only a few studies that investigated the impact of emotional stimuli on working memory performance, but their usefulness for the present questions is limited: these studies included either only younger adults or negative stimuli (Kensinger and Corkin, 2003) or examined the effect of arousal but not emotion (Wurm et al., 2004). The literature on attention and the positivity bias suggests that the pattern may be complicated. For instance, Allard and Isaacowitz (2008) found that older adults fixated their gaze more on positive and neutral images compared to negative images, regardless of whether the images were presented in full or divided attention conditions; additionally, older adults’ secondary task performance was as good as that of younger adults. This finding strongly suggests that processing positive emotional material does not require full attention. Other studies, however, have found that the presence of a distractor task leads older adults to remember proportionally more negative images than positive images (Mather and Knight, 2005) and to exhibit an attentional preference for negative images relative to positive images (Knight et al., 2007). This set of findings then indicates that processing positive emotional material does require attention, and that the age-related positivity bias may disappear or reverse under conditions of high cognitive load. Our study could shed further light on this controversy. That is, if the cognitive or memory load explanation has any validity, we would expect an age-related positivity effect for the *N*-Back conditions with low cognitive load (i.e., 0-Back or 1-Back), but a reversal of the effect for the 2-Back version, which has an added memory component which has been shown to increase response time (RT) and decrease accuracy (e.g., Verhaeghen and Basak, 2005).

We additionally recorded changes in participants’ pupil diameter during stimulus processing. The aim was to investigate how this response changes with age and/or as a function of processing demands and emotional content of the stimuli. Pupil dilations have long been thought to reflect a brain activity during perceptual and cognitive processing (Kahneman and Beatty, 1966). Task-evoked pupillary responses occur during the processing of stimuli and have been interpreted as indicators of cognitive effort and emotional arousal (for a review, see Beatty, 1982; Goldinger and Papesh, 2012). In line with this reasoning, pupil diameter typically increases as task demand increases (e.g., Karatekin et al., 2007; Van Gerven et al., 2004; Heitz et al., 2008) and as task/response interference increases (e.g., Porter et al., 2007; Laeng et al., 2011). Pupils also dilate during emotional processing, with larger pupil dilations occurring in response to positively valenced images (Hess, 1965), sexually arousing stimuli (Hicks et al., 1967), and positively and negatively arousing sounds (Partala and Surakka, 2003).

Surprisingly, few studies have investigated the joint influence of processing demands and emotional content on task-evoked pupillary responses. One exception is Stanners et al. (1979) who manipulated both processing demands (difficulty level of an arithmetic task) and arousal (threat of shock) or only arousal without processing demands. They found that pupillary responses increased with the difficulty of the arithmetic problems regardless of threat of shock, suggesting that pupillary responses are influenced mainly by cognitive demands of the task regardless of emotional arousal. In the low-difficulty task, however, arousal did influence pupil dilations. The two results combined suggest that arousal influences pupillary responses in low-demand situations, but that high cognitive demand may override the arousal-related response.

Based on this literature, we propose that pupil dilation during stimulus processing (relative to a baseline pupil measurement) could provide an independent, physiological indication of age-related differences in the effects of working memory demands (by comparing responses in the 2-Back task with responses in the 0-Back and 1-Back tasks) and potentially also of age-related differences in phasic changes due to emotion-specific arousal; the latter effect will likely only be observable in the low-demand conditions (i.e., 0-Back and 1-Back), as observed by Stanners et al. (1979).

To summarize, in the present study, we investigate one potential source of the oft-noted age-related differences in working memory performance, namely the possibility that older adults are less flexible than younger adults in unbinding information in working memory when representing the bound object is unnecessary – the so-called hyper-binding hypothesis. To maximize the chances of hyper-binding, we used emotional faces as stimuli; it has been shown that extracting the emotion content from such faces is an effortful process. The task is a subject-paced *N*-Back task. In its side-by-side 0-Back form, this task measures perceptual discrimination; its 1-Back version adds an attentional/STM requirement; the 2-Back version adds a working memory component. If older adults do hyper-bind, they would need more time and/or be less accurate in unbinding a stimulus into its features. As a consequence, we would expect larger age differences in the condition in which subjects respond only to the emotional expression. Additionally, we investigated the role of emotional valence on age differences in perception, attention, and memory, expecting, from the one extant study (Knight et al., 2007), a positivity bias in perception and attention (i.e., in 0-Back and 1-Back) and a negativity bias in working memory (i.e., in 2-Back), possibly (Knight et al., 2007) modulated by task difficulty such that the positivity bias would only show in the simpler tasks (i.e., 0-Back or 1-Back). Pupil dilation data will provide information as to the resource investment in each of the conditions. Given previous reports of age differences in performance in *N*-Back tasks, we expect that older adults would show a higher investment of resources in this task than younger adults.

The present work breaks modest new ground in at least two aspects. First, as far as we know, although emotional faces have been used extensively in perceptual paradigms such as stimulus discrimination or classification, there are fewer data on how working memory handles this class of stimuli (for one exception, see Kensinger and Corkin, 2003), and none in the field of aging. Second, as far as we know, we are the first research team to employ a physiological measure, pupil dilation, as independent verification of adult age-related differences in the task difficulty presumably involved in working memory encoding and/or retrieval and in the processing of emotional content.

## MATERIALS AND METHODS

### PARTICIPANTS

Twenty-one younger (67% female) and 21 older (71% female) adults participated in this experiment. Older participants were recruited from the community; they received cash payment ($10/hour) as compensation for participation. Younger adults were students at Georgia Institute of Technology; they were given either course credit or cash payment ($10/hour) for participation.

One older adult whose data could not be recorded due to technical problems was excluded from the analyses. The mean age of the remaining 20 older adults was 70.55 (*SD* = 4.3); mean age of younger adults was 20.33 (*SD* = 1.62). Older adults (*M* = 16.7, *SD* = 3.16) had completed more years of education than younger adults (*M* = 14.21, *SD* = 1.49), *t*(39) = 3.25, *p* = 0.002. Younger adults (*M* = 62.9, *SD* = 8.04) performed significantly better than older adults (*M* = 45.65, *SD* = 8.57) on a symbol digit test, *t*(39) = 6.65, *p* < 0.001. On the other hand, older adults’ performance (*M* = 34.1, *SD* = 4.51) on the Shipley Vocabulary test was significantly higher than performance of younger adults (*M* = 31.33, *SD* = 3.61), *t*(39) = 2.18, *p* = 0.04.

### MATERIALS

#### Faces

**FIGURE 1:**
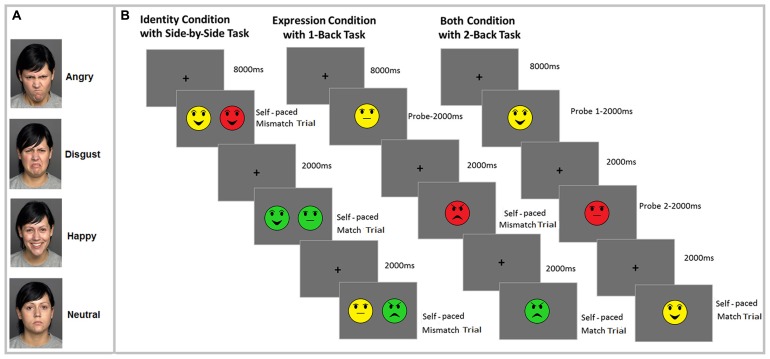
**Illustration of the one of the faces with all emotional expressions (angry, disgust, happy, neutral) used in the study (A) and the experimental procedure, with a sample *N*-Back task for each of the three conditions: identity (side-by-side 0-Back task), expression (1-Back task) and binding (2-Back task) (B).** Note that different colors represent different individual faces.

Forty-eight faces were selected from the FACES database (Ebner et al., 2010), with 12 different young-female actors portraying either angry, happy, neutral, or disgusted expressions. One of the faces, showing all these emotions is presented in **Figure 1A**. To keep the number of to-be-analyzed variables manageable we included only young and female faces in the study. Previous research has shown effects of age (young vs. old) and sex (female vs. male) of face stimuli on attention and memory performance of younger and older adults (for a meta-analytic review, see Rhodes and Anastasi, 2012); however, investigation of the effect of these variables is out of the scope of the current study.

Our choice of emotional expressions was guided by previous findings concerning age-related differences in emotion processing; the aim was to bias the results toward observing age by condition interactions. We included happy and angry faces specifically because older adults have been shown to show high recognition accuracy for happy stimuli (e.g., Carstensen et al., 1999) while younger adults tend to show a memory bias for angry faces (Mikels et al., 2005). Additionally, we included disgust to see whether recognition of disgust appears to be relatively preserved in old age as shown in a couple of studies (e.g., Calder et al., 2003).

We selected a subset of 12 young-female faces with happy, angry, neutral, and disgusted expressions from the FACES database based on correct identification of these facial expressions by younger-female, younger-male, older-female, and older-male raters in the norming study (Ebner et al., 2010)^1^. Given that we were interested in the effects of emotion alone, arousal level of the stimuli is a potential confound. Since arousal ratings are not available in FACES database, we conducted a separate study including 42 young adults (55% female) recruited from the Georgia Institute of Technology (mean age 19.81, *SD* = 2.12). Participants provided arousal ratings for each face on a 9-point Likert scale (1 – *not arousing at all*, 9 – *highly arousing*) using the Self-Assessment Manikin (Bradley and Lang, 1994). As expected, participants rated neutral faces (*M* = 3.13, *SD* = 1.42) as less arousing than happy (*M* = 5.26, *SD* = 1.56), disgusted (*M* = 6.00, *SD* = 1.73), or angry faces (*M* = 5.56, *SD* = 1.53), all *t*s(41) > 6.35, *p*s < 0.001. Although the perceived arousal level of individual face pictures in each emotion category varied, disgusted and angry faces were on average not more arousing than happy faces. The only significant difference was between angry and disgusted faces, *t*(41) = 4.41, *p* < 0.001.

#### Apparatus

Pupil data were recorded binocularly using a head-mounted SR Inc. Eyelink II eyetracker with a 500-Hz sample rate. To obtain baseline pupil estimates, an 8,000 ms fixation cross was presented at the beginning of each block. For each block, the average pupil size recorded during this 8,000 ms period served as the baseline pupil estimate for that block. Peak pupil sizes were recorded during presentation of the face photos and during the interstimulus interval. For the analyses, the peak pupil size value during stimulus presentation and the interstimulus interval for each trial was determined. The percentage change was calculated as max pupil size at each trial minus the baseline pupil value divided by the baseline, thus scaling responses as a function of each individual subject’s baseline (e.g., Iqbal et al., 2004).

All tasks were prepared and presented by E-Prime 2.2. Stimuli were presented on a 14-inch computer screen (resolution 1024 × 768 pixel) over a gray background (RGB: 150, 150, 150) as bitmap images (231 × 185 pixels). The face stimuli subtended a horizontal visual angle of 7.5° and a vertical visual angle of 6.6°.

#### Task design

The experiment consisted of identity, expression, and both conditions with three *N*-Back tasks (side-by-side 0-Back, 1-Back, and 2-Back). The task design for each condition is illustrated in **Figure 1B**^2^. Each condition involved making match/mismatch judgments based on either the identity of the face (identity condition), the face’s emotional expression (expression condition), or joint identity and expression of the faces (both condition). Each condition consisted of two blocks of 48 trials of each *N*-Back task. Since we had three different *N*-Back tasks, there were a total of 288 trials in each of the three conditions (identity, expression, binding). The number of match and mismatch trials was equally distributed across the 48 trials within each block. Furthermore, different trials included equal number of difficult and easy lures in each *N*-Back tasks. For instance, when participants were shown the face of person “A” with happy facial expression in the identity condition, the face of person “B” with happy facial expression was considered a difficult lure, whereas the face of person “B” with angry facial expression was an easy lure. Within each condition and task, the same set of face stimuli were used. This was done to ensure that performance differences between the different tasks and conditions could not be attributable to the stimuli. Emotional expressions and facial identity were pseudo-randomized so that each face and emotional expression was presented an equal number of times within each *N*-Back task. Conditions were blocked. The order of presentation of *N*-Back tasks and conditions was counterbalanced using a Latin square design. There was no effect of counterbalancing on performance.

#### Side-by-side 0-Back task

For the side-by-side 0-Back task, two face pictures were presented side-by-side on the screen and participants decided whether the two pictures were identical or not in terms of either identity or emotional expression, or of both identity and emotional expression, depending on condition. The purpose of the side-by-side task was to evaluate potential age differences in perceptual discrimination of facial identity, emotional expression, and their conjunction. Note that this side-by-side comparison likely involves saccadic eye movements, making it likely that the RTs for the 0-Back task will be larger than those of the 1-Back and 2-Back tasks.

#### 1-Back and 2-Back tasks

In these tasks, participants were asked to compare either identity, emotional expression, or joint identity and emotional expression of a face with the face presented one position back (1-Back) or two positions back (2-Back task).

### PROCEDURE

Participants were tested individually. Before the experiment, each participant signed a consent form. Each condition began with 12 practice trials. During the study, the eye tracker was calibrated before each block in each condition to establish a map between each participant’s gaze position and the eye tracker. Each block began with an 8,000 ms presentation of a fixation cross that served to collect the baseline pupil measurement. Next, participants were presented with a probe face (1-Back) or two probe faces (2-Back) for 2,000 ms each, or no probe (side-by-side 0-Back task). Following probe trials, participants completed 48 self-paced trials. In each trial, a single face (1-Back and 2-Back conditions) or two side-by-side faces (0-Back condition) were presented; subjects indicated whether the relevant feature(s) of the presented face matched the relevant feature(s) of the face presented one face back (1-Back condition), two faces back (2-Back condition), or whether the relevant feature(s) of the two side-by-side faces matched (0-Back). Because face trials were self-paced and peak pupil dilation typically occurs 1,800–2,000 ms post-stimulus onset (Beatty, 1982), each face trial was followed by a 2,000 ms interstimulus interval to allow sufficient time for the pupil to reach peak dilation. After 48 trials, a new block began. Pupil dilation was continuously recorded throughout the task. The total duration of the experiment was approximately 120 min.

## RESULTS

### CORRECTED RECOGNITION RATES

**FIGURE 2:**
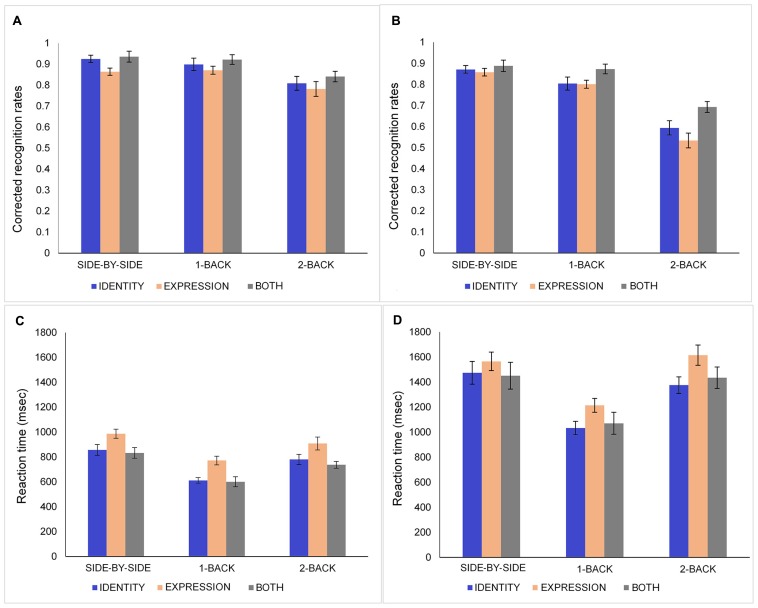
**Corrected recognition rates (proportion of hits minus false alarms) and response times of younger (A,C) and older (B,D) adults in the identity, expression, and both conditions for side-by-side 0-Back, 1-Back, and 2-Back tasks**. Error bars denote standard error.

Corrected recognition rates (proportion of hit rates minus false alarm rates) were calculated for each participant and entered in a three-way mixed analysis of variance (ANOVA): 2 (Age: young, old) × 3 (Condition: identity, expression, both) × 3 (*N*-Back: side-by-side 0-Back, 1-Back, 2-Back). The data for younger and older adults are presented in **Figures 2A,B**, respectively. Younger adults (*M* = 0.87, *SD* = 0.08) performed significantly better than older participants (*M* = 0.77, *SD* = 0.08), *F*(1,39) = 18.74, *MSE* = 0.05, *p* < 0.001, $\eta_{p}^{2}$ = 0.33. There was a main effect of condition [*F*(2,78) = 9.87, *MSE* = 0.02, *p* < 0.001, $\eta_{p}^{2}$ = 0.2] showing that participants performed better in the both (*M* = 0.86, *SD* = 0.11) than in the expression condition (*M* = 0.79, *SD* = 0.10), *t*(40) = 4.44, *p* < 0.001. Performance in the identity condition (*M* = 0.82, *SD* = 0.12) was not significantly different from performance in the expression and both conditions (all *p*s > 0.10). The main effect of *N*-Back task [*F*(2,78) = 134.58, *MSE* = 0.01, *p* < 0.001, $\eta_{p}^{2}$ = 0.78] revealed that participants had higher corrected recognition responses in the side-by-side 0-Back task (*M* = 0.89, *SD* = 0.07) than in the 1-Back task (*M* = 0.86, *SD* = 0.09), *t*(40) = 2.9, *p* = 0.006, and 2-Back task (*M* = 0.71, *SD* = 0.15), *t*(40) = 9.22, *p* < 0.001. Performance was higher in the 1-Back task than 2-Back task, *t*(40) = 9.89, *p* < 0.001.

Additionally, the ANOVA revealed a significant Age × *N*-Back interaction [*F*(2,78) = 27.65, *MSE* = 0.01, *p* < 0.001, $\eta_{p}^{2}$ = 0.42]. Follow-up analyses indicated that there was no age difference in the side-by-side task. Older adults performed significantly worse than younger adults in the 1-Back [*t*(39) = 2.79, *p* = 0.008] and 2-Back tasks [*t*(39) = 5.89, *p* < 0.001]. Moreover, while both age groups performed poorer in the 2-Back task than in the 1-Back and 0-Back versions, only older adults showed significantly poorer performance in the 1-Back compared to the 0-Back version [*t*(19) = 3.02, *p* = 0.007]. To examine the unbinding cost directly, a separate ANOVA: 2 (Age: young, old) × 2 (Condition: expression, both) × 3 (*N*-Back: side-by-side 0-Back, 1-Back, 2-Back) showed a significant Condition × *N*-Back × Age interaction [*F*(2,78) = 4.34, *MSE* = 0.01, *p* = 0.016, $\eta_{p}^{2}$ = 0.10]^3^. In the side-by-side 0-Back task, there was no age difference in the expression and both conditions. With regard to 1-Back task, there was no age difference in the both condition, but older adults performed worse than younger adults in the expression condition [*t*(39) = 2.59, *p* < 0.05]. For 2-Back task, older adults performed worse than younger adults in the expression [*t*(39) = 4.99, *p* < 0.001] and both [*t*(39) = 4.12, *p* < 0.001] conditions. More importantly, within the 2-Back task, older adults’ performance in the both condition exceeded that in the expression condition [*t*(19) = 3.25, *p* = 0.004]. However, younger adults did not show such difficulty in the expression condition compared to the both condition in the 2-Back task. For younger adults, the only difference between expression and both condition was within the side-by-side 0-Back task [*t*(20) = 3.09, *p* = 0.006]; their side-by-side 0-Back task performance was better in the both condition than the performance in the expression condition.

### RESPONSE TIMES

Any RTs 3 *SD* above or below the group mean values were excluded from the analyses. To eliminate the effect of age-related slowing in age by condition interaction analyses (Faust et al., 1999), a logarithmic transformation was applied on the RT data prior to analysis. Only hit trials were included in RT data analyses. The data for younger and older adults are presented in **Figures 2C,D**, respectively. A three-way mixed ANOVA on log transformed RTs showed that younger participants (*M* = 787.37, *SD* = 172.69) had faster reaction times than older participants (*M* = 1359.93, *SD* = 348.34), *F*(1,39) = 114.79, *MSE* = 0.22, *p* < 0.001, $\eta_{p}^{2}$ = 0.75. There was a main effect of task [*F*(2,78) = 122.66, *MSE* = 0.03, *p* < 0.001, $\eta_{p}^{2}$ = 0.76]. Participants had significantly longer RTs in side-by-side 0-Back task (*M* = 1194.7, *SD* = 296.13) than they had in 1-Back (*M* = 884.02, *SD* = 219.95), *t*(40) = 15.86, *p* < 0.001 and 2-Back tasks (*M* = 1142.22, *SD* = 265.45), *t*(40) = 3.72, *p* = 0.001. RTs were longer in 2-Back task than in 1-Back task [*t*(40) = 11.76, *p* < 0.001]. Longer RTs in the side-by-side task are consistent with participants’ verbal reports that they looked for differences in fine details between the two pictures; it is also likely that this RT included saccadic eye movements. ANOVA revealed a significant main effect of condition, *F*(2,78) = 17.58, *MSE* = 0.05, *p* < 0.001, $\eta_{p}^{2}$ = 0.31. Participants had longer RTs in the expression (*M* = 1176.93, *SD* = 250.89) condition than in the identity [*M* = 1022.43, *SD* = 237.28; *t*(40) > 6.26, *p* < 0.001] and both conditions [*M* = 1021.58, *SD* = 293.37; *t*(40) = 5.01, *p* < 0.001].

### INFLUENCE OF EMOTION ON ACCURACY

The analysis of the effect of emotional expression was restricted to the 1-Back and 2-Back tasks (the side-by-side comparison in the 0-Back task makes it impossible to assess which of the emotional expressions influenced performance), and to the expression and both conditions (in the identity condition, participants were supposed to disregard emotional information, and therefore any calculation of accuracy for expression is not meaningful in this condition).

**FIGURE 3:**
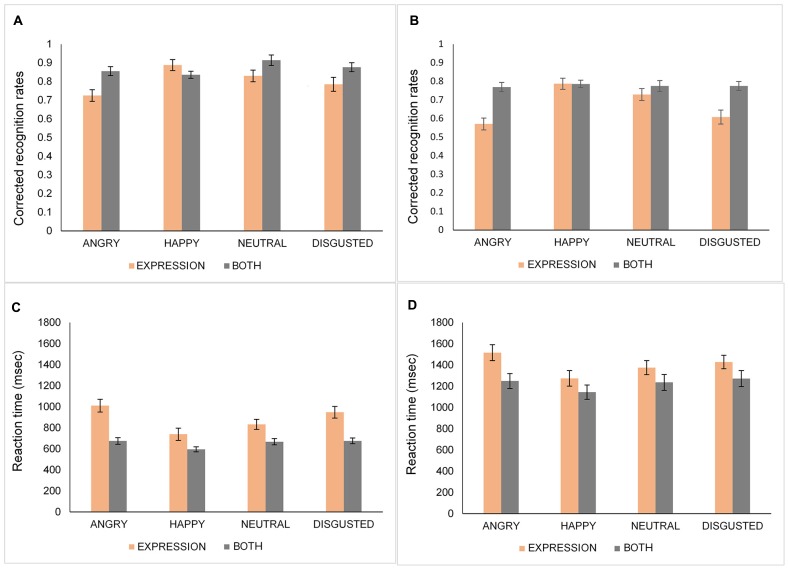
**Corrected recognition rates (proportion of hits minus false alarms) and response times of younger (A,C) and older (B,D) adults for angry, happy, neutral, and disgusted faces in the identity, expression, and both conditions**. Error bars denote standard error.

A four-way mixed ANOVA: 2 (Age: young, old) × 2 (Condition: expression, both) × 2 (*N*-Back: 1-Back, 2-Back) × 4 (Emotion: happy, angry, neutral, disgust) was performed on corrected recognition rates (proportion of hit minus false alarm rates) for each emotional expression. The data for younger and older adults are represented in **Figures 3A,B**, respectively. The main effect of emotion [*F*(3,117) = 18.87, *MSE* = 0.02, *p* < 0.001, $\eta_{p}^{2}$ = 0.33] showed that happy (*M* = 0.83, *SD* = 0.09) and neutral (*M* = 0.81, *SD* = 0.12) faces were recognized better than angry faces (*M* = 0.73, *SD* = 0.11) [happy vs. angry, *t*(40) = 6.86, *p* < 0.001; neutral vs. angry, *t*(40) = 5.82, *p* < 0.001]. Additionally, disgusted faces (*M* = 0.76, *SD* = 0.13) were recognized significantly worse than happy [*t*(40) = 3.93, *p* < 0.001], and neutral faces [*t*(40) = 3.21, *p* = 0.003]. There was no significant difference between angry and disgusted faces [*t*(40) = 2.04, *p* = 0.048] and between happy and neutral faces [*t*(40) = 0.99, *p* > 0.10]. Furthermore, the Condition by Emotion interaction [*F*(3,117) = 17.85, *MSE* = 0.02, *p* < 0.001, $\eta_{p}^{2}$ = 0.32] was significant. Follow-up analyses showed that in the expression condition, happy faces were recognized better than angry [*t*(40) = 8.39, *p* < 0.001], disgusted [*t*(40) = 5.62, *p* < 0.001] and, neutral faces [*t*(40) = 3.21, *p* = 0.003] while there was no difference in recognition of the faces in the both condition [all *t*s(40) < 2.05, *p*s > 0.09]. Additionally, participants performed better in the both condition than in the expression condition for angry [*t*(40) = 6.3, *p* < 0.001], and disgusted [*t*(40) = 4.48, *p* < 0.001] faces. There was no age-related recognition bias for the specific type of emotional stimuli shown, as indexed by a non-significant interaction between age and emotion [*F*(3,117) = 1.83, *MSE* = 0.02, *p* > 0.10, $\eta_{p}^{2}$ = 0.05].

### INFLUENCE OF EMOTION ON RESPONSE TIMES

The data for younger and older adults are shown in **Figures 3C,D**, respectively. The main effect of emotion was significant [*F*(3,117) = 15.79, *MSE* = 0.09, *p* < 0.001, $\eta_{p}^{2}$ = 0.29]. Participants’ RTs were significantly faster for happy (*M* = 937.87, *SD* = 55.67) faces than for angry [*M* = 1112.47, *SD* = 59.21; *t*(40) = 6.58, *p* < 0.001], disgusted [*M* = 1080.38, *SD* = 55.35; *t*(40) = 4.36, *p* < 0.001], or neutral faces [*M* = 1027.71, *SD* = 54.6; *t*(40) = 3.52, *p* = 0.001]. In addition, neutral faces were detected faster than angry faces [*t*(40) > 3.9, *p* < 0.001]. Moreover, the Condition × Emotion interaction was significant [*F*(3,117) = 3.72, *MSE* = 0.07, *p* = 0.01, $\eta_{p}^{2}$ = 0.09]. Follow-up analyses showed that in the expression condition, happy faces were recognized faster than angry [*t*(40) = 7.55, *p* < 0.001], disgusted [*t*(40) = 4.73, *p* < 0.001], or neutral faces [*t*(40) = 4.58, *p* < 0.001]. Additionally, neutral faces were recognized faster than angry faces [*t*(40) = 4.73, *p* < 0.001]. For happy faces, there was no RT difference between conditions whereas the expression condition yielded slower RTs than the both condition for angry [*t*(40) = 6.94, *p* < 0.001], neutral [*t*(40) = 3.46, *p* = 0.001], and disgusted faces [*t*(40) = 4.22, *p* < 0.001]. Consistent with the accuracy data, a non-significant Age × Emotion interaction [*F*(3,117) = 1.94, *MSE* = 0.09, *p* > 0.10, $\eta_{p}^{2}$ = 0.05] reflects an absence of age-related speed bias for any type of emotional expression.

### INFLUENCE OF EMOTION AND COGNITIVE LOAD ON PUPIL RESPONSES

Prior to analysis, pupil data with missing observations due to eyeblinks or signal loss were discarded. Additionally, pupil values above 2.5 *SD* from the average of their 10 immediate neighbors were removed within each individual and replaced by linear interpolation, in accordance with guidelines by Goldinger et al. (2009). This procedure resulted in fewer than 4% corrected trials per participant. For the analyses, the max peak pupil size value during stimulus presentation and the interstimulus interval for each trial was determined. The percentage change was calculated as max pupil size at each trial minus the baseline pupil value divided by the baseline (e.g., Iqbal et al., 2004). Baseline pupil size was the average pupil size recorded during this 8,000 ms fixation cross presented at the beginning of each block. Only hit responses were included in the analyses.

**FIGURE 4:**
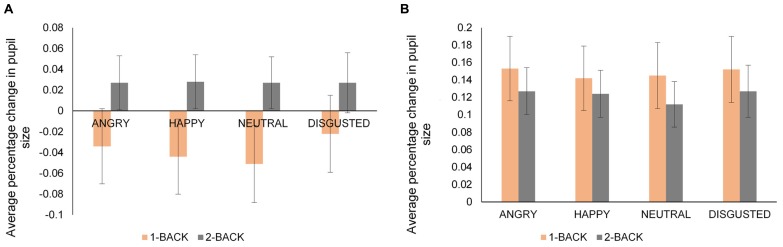
**Average percentage change in pupil size from a fixation-cross baseline for younger (A) and older (B) adults for angry, happy, neutral, and disgusted faces in 1-Back and 2-Back tasks**. Error bars denote standard error. Note the different scale of the vertical axis for younger and older adults.

To understand the joint effects of emotion and cognitive task on pupil size, average percentage change in pupil size was calculated for each emotional expression within the 1-Back and 2-Back versions of the task. As in the previous analyses focusing on emotion, side-by-side tasks in each condition and identity condition as a whole were not included. A four-way mixed ANOVA: 2 (Age: young, old) × 2 (Condition: expression, both) × 2 (*N*-Back: 1-Back, 2-Back) × 4 (Emotion: angry, happy, neutral, disgust) was performed on average percentage change in pupil size. Only hit trials were considered. The data for younger and older adults are presented in **Figures 4A,B**, respectively.

There was a main effect of emotion [*F*(3,117) = 6.77, *MSE* = 0.00, *p* < 0.001, $\eta_{p}^{2}$ = 0.15) with larger pupil dilation for disgusted and angry faces than neutral faces [disgusted vs. neutral faces, *t*(40) = 3.46, *p* = 0.001; angry vs. neutral faces *t*(40) = 3.37, *p* = 0.001]. More importantly, we obtained a significant N-Back × Emotion × Age interaction [*F*(3,117) = 3.55, *MSE* = 0.00, *p* = 0.02, $\eta_{p}^{2}$ = 0.08]. To follow-up, we ran separate ANOVAs on *N*-Back and Emotion within the groups of younger and older adults. Younger adults’ pupil dilation was larger in 2-Back task than 1-Back task [*F*(1,20) = 9.32, *MSE* = 0.00, *p* = 0.006, $\eta_{p}^{2}$ = 0.32] while older adults did not show a significant main effect of *N*-Back task [*F*(1,19) = 0.22, *MSE* = 0.00, *p* > 0.10, $\eta_{p}^{2}$ = 0.01]. For younger adults, pupil dilation was larger for angry and disgusted faces than neutral and happy faces in 1-Back task [angry vs. neutral, *t*(20) = 3.56, *p* = 0.002; disgust vs. neutral, *t*(20) = 3.33, *p* = 0.003; disgust vs. happy, *t*(20) = 3.28, *p* = 0.004]. However, there was no differential effect of any emotion on pupil dilation in 2-Back task for younger adults. These findings indicated that the effect of emotion on pupil dilation was evident only under low cognitive demand condition (in 1-Back task) for younger adults. On the other hand, the *N*-Back × Emotion interaction was not significant for older adults, showing that older adults’ pupil dilation was not different for the faces across *N*-Back tasks.

## DISCUSSION

Before we discuss the age-related effects in the data, we first turn to the general-psychological findings.

First, we indeed obtained the predicted difficulty effects for our *N*-Back tasks. Crucial for our main investigation, we found that the 1-Back task, which involves sequential comparisons between two stimuli, and is thus primarily a measure of attention and STM, is easier (i.e., it leads to higher accuracy and faster RT) than the 2-Back task, which involves retrieving a memory presentation stored outside the immediate focus of attention, and is thus primarily a measure of working memory performance. In older adults, the 0-Back task, which involves a side-by-side comparison and is thus primarily a measure of perceptual discrimination, was also easier (i.e., it led to higher accuracy) than the 1-Back task; younger adults showed statistically indistinguishable accuracy for the two tasks. Note that RT for the 0-Back task is much higher than for the other tasks. This is probably due to the peculiarity of our set-up: the two stimuli are presented side-by-side, likely prompting saccadic eye movements during the comparison process.

Second, our results concerning binding and unbinding of identity and emotion in the face stimuli are in line with expectations as well. Analysis of RTs showed that the identity condition (where only the identity of the face needed to be matched) and the both condition (where both identity and emotional expression needed to be matched) were statistically indistinguishable, and that they were performed on average about 150 ms faster than the expression condition (where only the emotional expression of the face needed to be matched). These results are in line with the literature on perceptual discrimination for emotional faces, where extraction of identity appears to happen relatively automatically, but extraction of emotional expression is an effortful, time-demanding process (e.g., Ganel and Goshen-Gottstein, 2004). The accuracy analyses added a subtle twist to these findings: subjects were more accurate in the both condition than in the expression condition, with the identity condition situated in between (and not significantly different from either). For younger adults (we will return to the older-adults data below), this effect is already present in the side-by-side 0-Back condition, and is simply carried over to the 1-Back and 2-Back versions of the task. This suggests that for younger adults the effect operates at the perceptual stage only – it is not further exacerbated by the attentional/STM requirement in the 1-Back, or the working memory requirement in the 2-Back task. In sum, the accuracy findings reaffirm that expression extraction is an added, effortful process, leading to lower accuracy, and suggest that identity extraction may come at a small (and, in the present study, not significant) cost in error rates.

The third set of findings concerns the effects of particular emotions on performance. This analysis was restricted to the 1-Back and 2-Back versions of the task (in mismatch stimuli in the 0-Back task, it is impossible to ascertain which of the two emotions displayed drives the effect); the analysis was also restricted to the two conditions were the emotional expressions were explicitly part of the decision process – the both condition and the expression condition. Overall, the evidence points at a positivity effect: happy and neutral faces yielded higher accuracy than angry and disgusted faces. This effect, however, only operated in the expression condition, not in the both condition, where no significant difference between accuracy for the different emotions was observed. RT data largely confirm this picture, with the exception of a less flat profile for the both condition, where happy faces were responded to faster than any other emotion. We note that the positivity effect in accuracy and RT may be due to the valence of the emotion, but it could also partially related to perceptual factors specific to happy faces, which tend to display more perceptually distinct features (i.e., open mouth, visible teeth) than negative and neutral faces. RTs in the both condition, with happy faces being the only stand-out (and being responded to fastest), lend some credence to the perceptual-distinctiveness hypothesis. Additionally, we showed that disgusted and angry emotions were more easily recognized in the both condition than in the expression condition. More accurate recognition of negative and potentially threatening emotions in the condition in which faces are processed as a whole may have its origin in the evolutionary survival value of swift action in the face of such emotions (e.g., Ackerman et al., 2006).

The final set of general findings pertains to pupil dilation. We highlight two results. First, for younger adults, we found evidence for a separation between the 1-Back task, which did not lead to larger pupil dilation compared to baseline, and the 2-Back task, which did. This finding is clearly in line with previous work showing that pupil dilation indexes cognitive resources (e.g., Granholm et al., 1996; Van Gerven et al., 2004; Goldinger and Papesh, 2012). Second, also for younger adults, but only for the 1-Back condition, we found a differential effect for emotion, with disgusted faces showing the highest amount of dilation, and angry the second highest. One observation here is that the emotions that yielded the lowest accuracy also elicit the largest pupil dilation, suggesting that higher effort (as evidenced by pupil dilation) is associated with more difficulties in maintaining attention across sequential presentation of stimuli. It also suggests that the recognition and reaction time advantage for happy faces is not due to attentional biases: one would expect that higher attentional effort would lead to better recognition and faster decisions, and therefore larger pupil dilation would be observed for happy faces. However, the opposite was true. We can solve this apparent puzzle if we assume that in the present case, pupil dilation does not index effort, but arousal (e.g., Stanners et al., 1979). In the separate study we conducted to assess arousal value of the stimuli (see Materials and Methods), the rank ordering of arousal values (from lowest to highest: neutral, happy, angry, disgusted) nicely tracks the pupil dilation date in the 1-Back condition for younger adults. We note that this differential effect of emotion disappeared in the 2-Back condition. The reason for this may be a functional ceiling in pupil dilation, in which the larger dilation associated with cognitive effort masks or trumps the arousal-related changes visible under conditions of low effort.

One goal of our study was to investigate age-related changes in the efficiency and/or efficacy of binding of information in working memory. Recall that binding emotional expressions to the identity of faces seems to be the default, and that abstracting emotional expressions from faces is effortful, as exemplified in higher accuracy and faster RTs for emotional faces than for the emotional expression on its own. Also recall that in younger adults, this effect operates at the perceptual stage, simply carrying over into the attentional/STM and working memory stages of processing (i.e., in the 1-Back and 2-Back task, respectively). The picture is different for older adults: age-related unbinding problems (operationalized as the difference between the expression condition and the both condition) become apparent in the 2-Back version of the task. Thus, older adults are as good (or as bad) as younger adults in unbinding the emotional feature from the compound stimulus in the perceptual and attentional/STM stages of the task; an added working memory component, however, widens the observed age difference. This suggests that older adults have a specific deficit, at least in this task, either with keeping unbound, feature-level information active and/or retrievable in memory, or with keeping the bound representation that is stored in working memory sufficiently detailed to easily abstract the emotional content from it. Further research is necessary to more precisely pinpoint the locus of or mechanism behind the effect. The result does not suggest a specific age-related problem of hyper-binding in the sense that older adults would keep the representation bound and younger adults do not: both age groups incur an unbinding cost in both accuracy and RT in the expression condition.

There is another possibility as well – an age-related deficit in flexible strategy use. Note that conditions were blocked, so that participants could freely apply strategic processing to the task, such as extraction of the relevant feature at the encoding stage rather than from a retrieved memory representation. If this strategy was used at all, the results suggest it was used more often, or with greater efficacy, by younger adults. In sum, the evidence points at the existence of hyper-binding in working memory in older adults, at least in this task, where the default modus operandi is to process stimuli in a bound state.

A second results concerns the emergence of age-related differences in the 1-Back task. In our previous work using the *N*-Back task (e.g., Verhaeghen et al., 2004; Vaughan et al., 2008), we consistently observed age-related differences in 2-Back tasks, but not in 1-Back tasks. The present experiment is the first where we do obtain age-related deficits in a 1-Back task. We can see at least two possible reasons for this difference. First, in our previous work, we used digits; the present study uses faces. Faces contain much more detail than digits, and so it is possible that this result signifies that older adults are less able to keep informational detail alive than younger adults do, even in the simple 1-Back task (for a related theoretical argument, see Myerson et al., 1990). The second possible reason is related to the brief interstimulus interval – 2,000 ms – built into the present, but not previous studies. The interpretation would then be that older adults lose informational detail at a faster clip than younger adults do. We are not aware of relevant data in visual working memory, but decay rates in verbal working memory are indeed faster in older than younger adults – the half-life of older adults’ STM representations is about 70% shorter than that of younger adults (Verhaeghen, 2014). Further research is necessary to assess to what degree each of those potential mechanisms operates to create the effect. We do know that this age-related deficit in attentional/STM processing cannot be due to perceptual deficits – younger and older adults performed equally well in the side-by-side comparison task.

Whatever the mechanism, it seems quite counterintuitive that age differences would appear with so minimal a cognitive load: all that is required of the subject in the 1-Back task is to retain an image of a face for 2 s, with no additional requirements. The pupil data confirm, however, that what one might consider a minimal load is far from minimal for older adults. First, older adults had elevated pupil dilations compared to younger adults in the 1-Back task, indicating that the 1-Back task is more effortful for older than for younger adults. Second, while we observed a clear separation between the 1-Back and the 2-Back version of the task in younger adults, older adults showed only nominally smaller pupil dilation for 1-Back that for 2-Back. This finding suggests that for older adults the cognitive load of passively maintaining a representation in the focus of attention is about as large as that for maintaining a representation in working memory while performing a concurrent task. One possible complication with the interpretation of this second finding is that there might be a functional ceiling for pupil dilation, and that statistical identity of 1-Back and 2-Back performance can therefore not directly equated with equal effort. Even under this interpretation, however, it is clear that the 1-Back task does not require attentional resources in younger adults, as testified by the lack of pupil dilation compared to baseline, whereas it clearly does in older adults.

A third age-related result concerns the differential effect of the emotion content of the stimuli. In general, and perhaps surprisingly, we observed a positivity effect, where happy and neutral faces were generally responded to faster and more accurately than angry and disgusted faces. Socioemotional selectivity theory (Carstensen et al., 1999) would predict this effect to be larger in older adults. One recent refinement of the theory (Mather and Knight, 2005) claimed that processing positive emotional material does require attention, and that the age-related positivity bias should then disappear or reverse under conditions of high cognitive load, as in our 2-Back task. We did not find any evidence for either hypothesis: no age by emotion interactions were observed in either accuracy or RTs, thus suggesting both an absence of an age-specific positivity effect and the absence of an effect of load on age-related difference in emotion-specific processing. Thus, the impact of the type of emotion was identical across age groups, regardless of processing stage (perceptual, attentional/STM, or memory) or cognitive load. This result runs counter socioemotional selectivity theory (including modifications of this theory that take cognitive load into account; e.g., Knight et al., 2007), but is in line with the one extant meta-analysis (Murphy and Isaacowitz, 2008) which found no significant difference in the preference for positive emotional stimuli of younger and older adults in either memory or attention studies.

Summarized, our results indicate that older adults do have more difficulty unbinding a bound stimulus than younger adults do, and that this difficulty is restricted to a working memory condition (2-Back); tasks primarily measuring perceptual clarity and attention/STM do not show age-related hyper-binding. Additionally, we found that, in general, older adults show deficits in attentional/STM processing (1-Back) as well as working memory processing; pupil dilation data suggest that attentional processing mobilizes and possibly requires more cognitive resources in older adults compared to younger adults. Finally, and importantly, our study adds to the body of literature suggesting a lack of age sensitivity in differential effects of different emotional stimuli on perception, attention, and working memory, counter the so-called positivity effect posited by socioemotional selectivity theory.

We note some important limitations of our study. One potential issue concerns the stimulus set: we used only young, female (and white) faces. Given the potential for own-age biases in attention and/or memory (Rhodes and Anastasi, 2012), it might be advisable to replicate this work using older-adult faces as well. We reiterative that same-age biases in face discrimination or face memory would influence mean levels of accuracy, but there is no reason to expect that that would influence interactions involving age.

Likewise, we obtained arousal data for these stimuli only from younger adults. Given that our study included both younger and older adults, arousal ratings from a sample of older adults would have been useful to ensure that older adults did not perceive the emotional faces differently than younger adults. Although some face databases (e.g., The Karolinska Directed Emotional Faces by Goeleven et al., 2008) measured perceived arousal level of the emotional face stimuli in the norming study, to our knowledge none of the databases included older adults as raters in their norming studies. When we look at non-face stimuli, Grühn and Scheibe (2008) investigated age-related differences in perceived levels of IAPS pictures and found that older adults perceived negative pictures as being more arousing and positive pictures as being less arousing than young adults did. If this result were to generalize to faces, we would expect larger pupil dilation for angry and disgusted faces in older adults compared to younger adults, and perhaps pupil contraction for happy faces. This is not what was found. We do note that this limitation is likely moot in the present study: only younger adults showed modulation of their pupil responses that could be interpreted as arousal-mediated, and then only in the 1-Back condition. Older adults, as well as younger adults in the more demanding condition (2-Back), showed elevated pupil dilation and no modulation by emotion, suggesting that the cognitive demands of the task wiped out any arousal-based effects on pupil dilation. Of course, it remains theoretically possible that older adults perceived all faces as much more arousing than younger adults did, thereby in effect attenuating all differential effects of emotion on pupil dilation.

A third limitation is that we scaled our pupillometry data as percentage of baseline; recent work (unknown to us when we were designing the experiment) has used a different approach, scaling pupil size as a function of maximum possible pupil change (Piquado et al., 2010). We cannot retroactively apply this metric to our data, but this rescaling would have been useful to see if older adults’ asymptoting of pupil size in the 1-Back condition reflects a limit on the deployment of attentional/STM resources or a physiological limit on pupil size.

Many questions remain. For instance, it is not clear whether the unbinding deficit in old age is a specific deficit, or merely an instantiation of the complexity effect – age differences in perception and attention are magnified in memory tasks, with the larger age differences then observed in the more difficult task. Under most circumstances, binding would be more difficult (a finding in line with the results on associative deficits summarized in the Introduction), but in the present study, unbinding was more difficult, and hence we found evidence for hyper-binding in older adults. Likewise, it is unclear what exactly makes the 1-Back, attention-centered condition so hard and effortful for older adults: information detail, the 2-s delay, or some other mechanism. It would also be worthwhile to get neuroimaging data on the brain locus of this effect, and see whether it (as one would expect) originates in parts of the control network.

## Conflict of Interest Statement

The authors declare that the research was conducted in the absence of any commercial or financial relationships that could be construed as a potential conflict of interest.

## Footnotes

1 In the norm study (Ebner et al., 2010), for our subset of the stimuli, the mean accuracy of emotional expression identification by younger women was 93.15 (*SD* = 11.31), by younger men was 90.56 (*SD* = 10.91), by older women was 86.42 (*SD* = 15.17), and by older men was 83.67 (*SD* = 16.42).

2 Since the FACES database agreement provides only one young-female face to use in scientific publications, it was not possible to demonstrate the tasks we used in this study using the real stimuli. Thus, for the purposes of task design illustration, we used emoticons in **Figure 1B**.

3 When we conducted a separate ANOVA: 2 (Age: young, old) × 2 (Condition: identity, both) × 3 (*N*-Back: side-by-side 0-Back, 1-Back, 2-Back) to see whether age-related unbinding cost would occur for identity task, the Condition × *N*-Back × Age interaction was not significant, indicating no differential cost of unbinding for older adults in the identity condition.

## References

[B1] Ackerman J. M. Shapiro J. R. Neuberg S. L. Kenrick D. T. Becker D. V. Griskevicius V. (2006). They all look the same to me (unless they’re angry): from out-group homogeneity to outgroup heterogeneity. *Psychol. Sci.* 17 836–840 10.1111/j.1467-9280.2006.01790.x 17100781

[B2] Allard E. S. Isaacowitz D. M. (2008). Are preferences in emotional processing affected by distraction? Examining the age-related positivity effect in visual fixation within a dual-task paradigm. *Aging Neuropsychol. Cogn.* 15 725–743 10.1080/13825580802348562 PMC2645630 18819026

[B3] Baddeley A. D. Hitch G. J. (1974). “Working memory,” in *The Psychology of Learning and Motivation* eds Bower G. H. (New York: Academic Press) 47–89

[B4] Beatty J. (1982). Task-evoked pupillary responses, processing load, and the structure of processing resources. *Psychol. Bull.* 91 276–292 10.1037/0033-2909.91.2.276 7071262

[B5] Bopp K. L. Verhaeghen P. (2007). Age-related differences in control processes in verbal and visuo-spatial working memory: storage, transformation, supervision, and coordination. *J. Gerontol. B Psychol. Sci. Soc. Sci.* 62 239–246 10.1093/geronb/62.5.P239 17906164

[B6] Bopp K. L. Verhaeghen P. (2009). Working memory and aging: separating the effects of content and context. *Psychol. Aging* 24 968–980 10.1037/a0017731 20025410 PMC2805123

[B7] Bradley M. M. Lang P. J. (1994). Measuring emotion: the self-assessment manikin and the semantic differential. *J. Behav. Ther. Exp. Psychiatry* 25 49–59 10.1016/0005-7916(94)90063-9 7962581

[B8] Brockmole J. R. Parra M. A. Della Sala S. Logie R. H. (2008). Do binding deficits account for age-related decline in visual working memory? *Psychon. Bull. Rev.* 15 543–547 10.3758/PBR.15.3.543 18567252

[B9] Bruce V. Young A. W. (1986). Understanding face recognition. *Br. J. Psychol.* 77 305–327 10.1111/j.2044-8295.1986.tb02199.x 3756376

[B10] Calder A. J. Keane J. Manly T. Sprengelmeyer R. Scott S. Nimmo-Smith I. (2003). Facial expression recognition across the adult life span. *Neuropsychologia* 41 195–202 10.1016/S0028-3932(02)00149-5% 12459217

[B11] Campbell K. L. Hasher L. Thomas R. C. (2010). Hyper-binding: a unique age effect. *Psychol. Sci.* 21 399–405 10.1177/0956797609359910 20424077 PMC3399902

[B12] Carstensen L. L. Isaacowitz D. Charles S. T. (1999). Taking time seriously: a theory of socioemotional selectivity. *Am. Psychol.* 54 165–181 10.1037/0003-066X.54.3.165 10199217

[B13] Cerella J. (1985). Information processing rates in the elderly. *Psychol. Bull.* 98 67–83 10.1037/0033-2909.98.1.67 4034819

[B14] Chalfonte B. L. Johnson M. K. (1996). Feature memory and binding in young and older adults. *Mem. Cogn.* 24 403–416 10.3758/BF03200930 8757490

[B15] Chen T. Li D. (2007). The roles of working memory updating and processing speed in mediating age-related differences in fluid intelligence. *Neuropsychol. Dev. Cogn. B Aging Neuropsychol. Cogn.* 14 631–646 10.1080/13825580600987660 18038360

[B16] Conway A. R. A. Cowan N. Bunting M. F. Therriault D. J. Minkoff S. R. B. (2002). A latent variable analysis of working memory capacity, short-term memory capacity, processing speed, and general fluid intelligence. *Intelligence* 30 163–183 10.1016/S0160-2896(01)00096-4

[B17] Cowan N. Naveh-Benjamin M. Kilb A. Saults J. S. (2006). Life-span development of visual working memory: when is feature binding difficult? *Dev. Psychol*. 42 1089–1102 10.1037/0012-1649.42.6.1089% 17087544 PMC1635970

[B18] Dobbs A. R. Rule B. G. (1989). Adult age differences in working memory. *Psychol. Aging* 4 500–503 10.1037/0882-7974.4.4.500 2619956

[B19] Ebner N. Riediger M. Lindenberger U. (2010). FACES – a database of facial expressions in young, middle-aged, and older women and men: development and validation. *Behav. Res. Methods* 42 351–362 10.3758/BRM.42.1.351 20160315

[B20] Engle R. W. Kane M. J. Tuholski S. W. (1999). “Individual differences in working memory capacity and what they tell us about controlled attention, general fluid intelligence and functions of the prefrontal cortex,” in *Models of Working Memory: Mechanisms of Active Maintenance and Executive Control* eds Miyake A. Shah P. (London: Cambridge University Press) 102–134

[B21] Faust M. E. Balota D. A. Spieler D. H. Ferraro F. R. (1999). Individual differences in information-processing rate and amount: implications for group differences in response latency. *Psychol. Bull.* 125 777–799 10.1037/0033-2909.125.6.777 10589302

[B22] Ganel T. Goshen-Gottstein Y. (2004). Effects of familiarity on the perceptual integrality of the identity and expression of faces: the parallel-route hypothesis revisited. *J. Exp. Psychol. Hum. Percept. Perform.* 30 583–597 10.1037/0096-1523.30.3.583 15161388

[B23] Goeleven E. Raedt R. Leyman L. Verschuere B. (2008). The Karolinska directed emotional faces: a validation study. *Cogn. Emot.* 22 1094–1118 10.1080/02699930701626582

[B24] Goldinger S. D. Papesh M. H. (2012). Pupil dilation reflects the creation and retrieval of memories. *Curr. Dir. Psychol. Sci.* 21 90–95 10.1177/0963721412436811 PMC5662122 29093614

[B25] Goldinger S. D. He Y. Papesh M. (2009). Deficits in cross-race face learning: insights from eye-movements and pupillometry. *J. Exp. Psychol. Learn. Mem. Cogn.* 35 1105–1122 10.1037/a0016548 19686008 PMC2871406

[B26] Granholm E. Asarnow R. F. Sarkin A. J. Dykes K. L. (1996). Pupillary responses index cognitive resource limitations. *Psychophysiology* 33 457–461 10.1111/j.1469-8986.1996.tb01071.x 8753946

[B27] Grühn D. Scheibe S. (2008). Age-related differences in valence and arousal ratings of pictures from the International Affective Picture System (IAPS): do ratings become more extreme with age? *Behav. Res. Methods* 40 512–521 10.3758/BRM.40.2.512 18522062

[B28] Heitz R. P. Schrock J. C. Payne T. W. Engle R. W. (2008). Effect of incentive on working memory capacity: behavioral and pupillometric data. *Psychophysiology* 45 119–129 10.1111/j.1469-8986.2007.00605.x 17910734

[B29] Hess E. H. (1965). Attitude and pupil size. *Sci. Am.* 212 46–54 10.1038/scientificamerican0465-46 14261525

[B30] Hicks R. A. Reaney T. Hill L. (1967). Effects of pupil size and facial angle on preference for photographs of a young woman. *Percept. Mot. Skills* 24 388–390 10.2466/pms.1967.24.2.388 6040211

[B31] Iqbal S. T. Zheng X. S. Bailey B. P. (2004). “Task-evoked pupillary response to mental workload in human-computer interaction,” in *Proceedings of the Conference on Human Factors in Computing Systems* Vienna, Austria 2004 1477–1480 10.1145/985921.986094

[B32] Kahneman D. Beatty J. (1966). Pupil diameter and load on memory. *Science* 154 1583–1585 10.1126/science.154.3756.1583 5924930

[B33] Kane M. J. Bleckley M. K. Conway A. R. A. Engle R. W. (2001). A controlled-attention view of working memory capacity: individual differences in memory span and the control of visual orienting. *J. Exp. Psychol. Gen.* 130 169–183 10.1037/0096-3445.130.2.169 11409097

[B34] Karatekin C. Marcus D. J. Couperus J. W. (2007). Regulation of cognitive resources during sustained attention and working memory in 10-year-olds and adults. *Psychophysiology* 44 128–144 10.1111/j.1469-8986.2006.00477.x 17241149

[B35] Kemper S. Herman R. E. Lian C. H. T. (2003). The costs of doing two things at once for young and older adults: talking while walking, finger tapping, and ignoring speech or noise. *Psychol. Aging* 18 181–192 10.1037/0882-7974.18.2.181 12825768

[B36] Kensinger E. A. Corkin S. (2003). Effects of negative emotional content on working memory and long-term memory. *Emotion* 3 378–393 10.1037/1528-3542.3.4.378 14674830

[B37] Kirchner W. K. (1958). Age differences in short-term retention of rapidly changing information. *J. Exp. Psychol.* 55 352–358 10.1037/h0043688 13539317

[B38] Knight M. Seymour T. L. Gaunt J. T. Baker C. Nesmith K. Mather M. (2007). Aging and goal directed emotional attention: distraction reverses emotional biases. *Emotion* 7 705–714 10.1037/1528-3542.7.4.705 18039037

[B39] Kyllonen P. C. (1996). “Is working memory capacity Spearman’s g?,” in *Human Abilities: Their Nature and Measurement* eds Dennis I. Tapsfield P. (Mahwah, NJ: Erlbaum) 49–75

[B40] Laeng B. Ørbo M. Holmlund T. Miozzo M. (2011). Pupillary Stroop effects. *Cogn. Process.* 12 13–21 10.1007/s10339-010-0370-z 20865297 PMC3026931

[B41] Martens U. Leuthold H. Schweinberger S. R. (2010). On the temporal organization of facial identity and expression analysis: inferences from event-related brain potentials. *Cogn. Affect. Behav. Neurosci.* 10 505–522 10.3758/CABN.10.4.505 21098811

[B42] Mather M. Knight M. (2005). Goal-directed memory: the role of cognitive control in older adults’ emotional memory. *Psychol. Aging* 20 554–570 10.1037/0882-7974.20.4.554 16420131

[B43] Mikels J. A. Larkin G. R. Reuter-Lorenz P. A. Cartensen L. L. (2005). Divergent trajectories in the aging mind: changes in working memory for affective versus visual information with age. *Psychol. Aging.* 20 542–553 10.1037/0882-7974.20.4.542 16420130 PMC2746384

[B44] Miyake A. Shah P. (1999). “Toward unified theories of working memory: emerging general consensus, unresolved theoretical issues, and future research directions,” in *Models of Working Memory: Mechanisms of Active Maintenance and Executive Control* eds Miyake A. Shah P. (Cambridge: Cambridge University Press) 442–481 10.1017/CBO9781139174909

[B45] Murphy N. A. Isaacowitz D. M. (2008). Preferences for emotional information in older adults: a meta-analysis of memory and attention studies. *Psychol. Aging* 23 263–286 10.1037/0882-7974.23.2.263 18573002

[B46] Myerson J. Hale S. Wagstaff D. Poon L. W. Smith G. A. (1990). The information-loss model: A mathematical theory of age-related cognitive slowing. *Psychol. Rev.* 97 475–487 10.1037/0033-295X.97.4.475 2247538

[B47] Naveh-Benjamin M. Guez J. Kilb A. Reedy S. (2004). The associative deficit of older adults: further support using face-name associations. *Psychol. Aging* 19 541–546 10.1037/0882-7974.19.3.541 15383004

[B48] Oberauer K. (2002). Access to information in working memory: exploring the focus of attention. *J. Exp. Psychol. Learn. Mem. Cogn.* 28 411–421 10.1037/0278-7393.28.3.411 12018494

[B49] Old S. Naveh-Benjamin M. (2008). Differential effects of age on item and associative measures of memory: a meta-analysis. *Psychol. Aging* 23 104–118 10.1037/0882-7974.23.1.104 18361660

[B50] Partala T. Surakka V. (2003). Pupil size variation as an indication of affective processing. *Int. J. Hum. Comput. Stud.* 59 185–198 10.1016/S1071-5819(03)00017.x

[B51] Piquado T. Isaacowitz D. M. Wingfield A. (2010). Pupillometry as a measure of cognitive effort in younger and older adults. *Psychophysiology* 47 560–569 10.1111/j.1469-8986.2009.00947.x 20070575 PMC2867103

[B52] Porter G. Troscianko T. Gilchrist I. D. (2007). Effort during visual search and counting: insights from pupillometry. *Q. J. Exp. Psychol.* 60 211–229 10.1080/17470210600673818 17455055

[B53] Rhodes M. G. Anastasi J. S. (2012). The own-age bias in face recognition: a meta-analytic and theoretical review. *Psychol. Bull.* 138 146–174 10.1037/a0025750 22061689

[B54] Soto F. A. Wasserman E. A. (2011). Asymmetrical interactions in the perception of face identity and emotional expression are not unique to the primate visual system. *J. Vis.* 11:24 10.1167/11.3.24 21454855

[B55] Stanners R. Coulter M. Sweet A. Murphy P. (1979). The pupillary response as an indicator of arousal and cognition. *Motiv. Emot.* 3 319–340 10.1007/BF00994048

[B56] Van Gerven P. W. M. Paas F. Van Merriënboer J. J. G. Schmidt H. G. (2004). Memory load and the cognitive pupillary response in aging. *Psychophysiology* 41 167–174 10.1111/j.1469-8986.2003.00148.x 15032982

[B57] Vaughan L. Basak C. Hartman M. Verhaeghen P. (2008). Aging and working memory inside and outside the focus of attention: dissociations of availability and accessibility. *Aging Neuropsychol. Cogn.* 15 1–22 10.1080/13825580802061645 18608047

[B58] Verhaeghen P. (2012). “Working memory still working: age-related differences in working memory and executive control,” in *Memory and Aging* eds Ohta N. Naveh-Benjamin M. (New York: Psychology Press) 3–30

[B59] Verhaeghen P. (2014). *The Elements of Cognitive Aging: Meta-Analyses of Age-Related Differences in Processing Speed and Their Consequences*. New York, NY: Oxford University Press

[B60] Verhaeghen P. Basak C. (2005). Aging and switching of the focus of attention in working memory: results from a modified *N*-Back task. *Q. J. Exp. Psychol. A* 58 134–154 10.1080/02724980443000241 15881295

[B61] Verhaeghen P. Cerella J. Basak C. (2004). A working memory workout: how to change to size of the focus of attention from one to four in ten hours or less. *J. Exp. Psychol. Learn. Mem. Cogn.* 30 1322–1337 10.1037/0278-7393.30.6.1322 15521807

[B62] Wheeler M. E. Treisman A. M. (2002). Binding in short-term visual memory. *J. Exp. Psychol. Gen.* 131 48–64 10.1037/0096-3445.131.1.48 11900102

[B63] Wurm L. H. Labouvie-Vief G. Aycock J. Rebucal K. A. Koch H. E. (2004). Performance in auditory and visual emotional Stroop tasks: a comparison of older and younger adults. *Psychol. Aging* 19 523–535 10.1037/0882-7974.19.3.523 15383002

[B64] Zhang Y. Verhaeghen P. Cerella J. (2012). Working memory at work: how the updating process alters the nature of memory encoding. *Acta Psychol.* 139 77–83 10.1016/j.actpsy.2011.10.012 PMC3249472 22105718

